# Burden of CNS neoplasms in the Middle East and North Africa from 1990 to 2023, with projections to 2050: Insights from Global Burden of Disease

**DOI:** 10.1097/MD.0000000000051128

**Published:** 2026-10-16

**Authors:** Ibraheem Altamimi, Alaa Alanazi, Abdullah Abdulaziz Alnaim, Ahmed Khalid Alzahrani, Yousef Saeed Alzahrani, Abdulelah Mohammed Abdullah Alsakti, Norah Abdullah Alodhaib, Ali Abdulhakim Alhakeem, Mohammed Mastour Alharthi, Raghad Emad Alhalabi, Aalaa Saleh, Jaber Abdullah Alshahrani

**Affiliations:** a Dermatology Department, King Abdulaziz Medical City, Riyadh, Saudi Arabia; b Department of Medical Surgical Nursing, College of Nursing, Princess Nourah bint Abdulrahman University, Riyadh, Saudi Arabia; c King Faisal University, Al-Ahsa, Saudi Arabia; d College of Medicine, Shaqra campus, Shaqra University, Shaqra, Saudi Arabia; e College of Medicine, Jeddah University, Jeddah, Saudi Arabia; f King Khalid University, Abha, Saudi Arabia; g College of Medicine, Qassim University, Qassim, Saudi Arabia; h College of Medicine, King Faisal University, Hofuf, Saudi Arabia; i Ibn Sina National College, Jeddah, Saudi Arabia; j Batterjee Medical College (BMC), Asir, Saudi Arabia; k Faculty of Medicine, Lebanese University, Beirut, Lebanon; l Armed Forces Hospital Southern Region, Ministry of Defense Health Services, Khamis Mushait, Saudi Arabia.

**Keywords:** central nervous system neoplasms, incidence, Middle East and North Africa, mortality, prevalence

## Abstract

Central nervous system (CNS) neoplasms are uncommon but highly consequential malignancies that account for substantial neurological disability and premature mortality. The Middle East and North Africa (MENA) region is marked by rapid population growth, aging, and uneven health system capacity, yet comprehensive long-term evaluations of CNS neoplasm burden across countries, sexes, and age groups remain limited. We analyzed data from the Global Burden of Disease 2023 study to assess trends in incidence, prevalence, and mortality from CNS neoplasms in the MENA region from 1990 to 2023, with projections to 2050. Age-standardized incidence, prevalence, and mortality rates per 1,00,000 population were estimated. Temporal trends were evaluated using Joinpoint regression to calculate annual percent change and average annual percent change (AAPC) with 95% confidence intervals. Analyses were stratified by sex, age group, and country. Between 1990 and 2023, the age-standardized mortality rate remained largely stable with a small but significant upward trend (AAPC 0.09%). Mortality rates were consistently higher among males. Adults aged 20 years and older experienced rising age-standardized mortality (AAPC 0.58%), whereas mortality declined among individuals younger than 20 years (AAPC−0.90%). Incidence increased in both absolute numbers and age-standardized rates (AAPC 0.42%). Prevalence also rose steadily, with the age-standardized prevalence rate increasing from 13.46 to 19.51 per 1,00,000 (AAPC 1.13%), driven mainly by adults. The burden of CNS neoplasms in the MENA region has intensified over the past 3 decades, highlighting the need for improved surveillance, earlier diagnosis, and expanded access to specialized neuro-oncological care.

## 1. Introduction

Cardiovascular diseases are the leading cause of death worldwide, followed by cancer as the second most common cause, responsible for roughly 16 to 17 percent of all deaths.^[1,2]^ Primary brain and central nervous system cancers, referred to hereafter as CNS cancer, affect individuals across all age groups and can arise in any anatomical region of the CNS. More than 90 percent originate in the brain, with the remainder occurring in the meninges, spinal cord, and cranial nerves. Collectively, these malignancies contribute considerably to global morbidity and mortality.^[3]^ Gliomas are the most common type of CNS cancer, including the highly lethal subtype glioblastoma multiforme.^[3,4]^ CNS cancers vary in their characteristics according to the age of affected patients. Germ cell tumors occur more frequently in children and young adults, whereas malignant gliomas are more likely to develop with increasing age. In addition, indolent tumors often produce milder symptoms until they reach a critical size, while glioblastoma multiforme is marked by aggressive behavior, pronounced clinical manifestations, and invasion of adjacent structures.^[5,6]^ Consequently, CNS cancers show substantial variation not only in clinical presentation but also in their epidemiological patterns.

The Middle East and North Africa region (MENA) generally encompasses countries of the Arabian Peninsula such as Oman, Saudi Arabia, Kuwait, Qatar, the United Arab Emirates, Bahrain, and Yemen, along with Levantine states including Jordan, Lebanon, Palestine, Israel, and Syria. The region also commonly includes Egypt, Turkey, Iraq, Iran, and Cyprus.^[7]^

Few established risk factors are linked to CNS cancer. Epidemiological studies consistently show increased risk with exposure to ionizing radiation, such as atomic weapon exposure or prior therapeutic irradiation, and reduced risk among individuals with atopic conditions, including asthma, eczema, and food allergies.^[8,9]^ Several genetic syndromes and rare susceptibility alleles are linked to an increased risk of CNS cancer, but they explain only a very small proportion of cases.^[10,11]^ Greater insight into the geographic distribution of CNS cancer may help clarify the role of external factors in shaping its epidemiological patterns.

Earlier attempts to estimate the burden of CNS cancer have been restricted to individual countries or regions, focused on particular age groups or single-year assessments, or relied on outdated data.^[3,12,13]^ The MENA region has distinct epidemiological features that merit focused study, shaped by heterogeneous healthcare systems, wide economic variation, and rapidly changing population demographics. This study evaluated trends in the incidence, prevalence, and mortality of CNS neoplasms in the MENA region from 1990 to 2023 using data from the Global Burden of Disease (GBD) Study. We assessed differences by sex, age group, and country, and developed projections through 2050. The results aim to support evidence-based health planning and resource allocation in response to the changing burden of CNS neoplasms across the region.

## 2. Materials and methods

### 2.1. Overview

The GBD 2023 study builds on GBD 2021 and presents updated, systematic estimates for 204 countries and territories. It reports on 292 causes of death and includes measures of incidence, prevalence, mortality, years of life lost, and disability-adjusted life-years. Compared with GBD 2021, the 2023 iteration incorporates 11,474 additional data sources.^[14]^ All data were assembled by the Institute for Health Metrics and Evaluation (IHME), and all estimates follow the Guidelines for Accurate and Transparent Health Estimates Reporting (GATHER).^[15]^

### 2.2. Protocol approval and patient consent

Data for this study were retrieved from the Global Health Data Exchange (GHDx) query tool (http://ghdx.healthdata.org/gbd-results-tool), a publicly accessible, de-identified database. Because the data are aggregated and anonymized, institutional review board approval and informed consent were not required.

### 2.3. Disease definition

CNS neoplasms include malignant tumors of the meninges, brain, spinal cord, and cranial nerves. In the GBD framework, causes are structured into 4 hierarchical levels, starting with broad groupings at level 1, such as injuries, and progressing to specific conditions at level 4, such as hemorrhagic stroke. CNS neoplasms are designated as a level 3 cause within the broader level 2 category of neoplasms. Under the International Classification of Diseases, Tenth Revision (ICD-10), CNS neoplasms are defined by codes C70–C70.1 and C70.9–C72.9, whereas in ICD-9 they are classified under codes 191–191.9.^[14]^

### 2.4. Data sources and extraction

GBD data are derived from systematic reviews of vital registration systems, demographic censuses, and disease registries. In MENA countries, the main data sources were health insurance claims and encounter databases. Sex- and age-specific data on the incidence, prevalence, and mortality of CNS neoplasms were extracted for MENA populations.

### 2.5. Statistics analysis

Crude incidence, prevalence, and mortality rates for CNS neoplasms were calculated and age-standardized, with all rates reported per 1,00,000 population. Ninety 5 percent confidence intervals (CIs) were obtained from the 2.5th and 97.5th percentiles of 1000 ordered draws. Annual percentage changes in age-standardized incidence, prevalence, and mortality were estimated using the Joinpoint Regression Program, version 5.0.2 (National Cancer Institute).^[16]^ The model tested up to 5 joinpoints using Monte Carlo permutation methods^[17]^ to identify statistically distinct trend segments. The average annual percentage change was derived as a weighted mean of the segment specific estimates. Mortality trends through 2050 were projected using an autoregressive integrated moving average model trained on data from 1990 to 2021 and validated against observed values from 2021 to 2023 using time series cross validation. Model fit was evaluated with the Bayesian Information Criterion and root mean square error.^[18]^ Statistical significance was defined as *P* < .05.

## 3. Results

### 3.1. Mortality

#### 3.1.1. Overall and gender

Between 1990 and 2023, mortality from CNS neoplasms showed a substantial increase in absolute numbers alongside a largely stable age-standardized mortality rate (ASMR). A total of 5,97,728 deaths were recorded during the study period. Annual deaths rose from 10,856.45 (95% CI: 8023.82–14,749.90) in 1990 to 26,329.42 (95% CI: 20,624.60–34,267.37) in 2023, indicating a marked growth in the overall burden of deaths (Tables 1, 2 and Figs. S1, S2, Supplemental Digital Content 4). The ASMR increased slightly from 4.80 (95% CI: 3.49–6.92) per 1,00,000 in 1990 to 5.01 (95% CI: 3.96–6.61) per 1,00,000 in 2023, with a small but statistically significant upward trend (average annual percent change [AAPC] 0.09%*, 95% CI: 0.02–0.17; Table 3, Fig. 1). By sex, males consistently exhibited higher mortality rates than females. In 1990, the male ASMR was 5.30 (95% CI: 3.57–8.26), compared with 4.27 (95% CI: 2.93–6.70) among females. By 2023, ASMRs had increased modestly in both sexes to 5.54 (95% CI: 3.99–7.57) in males and 4.47 (95% CI: 3.06–6.31) in females. Trend analysis showed a stable pattern among males, with a nonsignificant AAPC of 0.13% (95% CI: 0.00–0.26), whereas females demonstrated a significant increase over time, with an AAPC of 0.13%* (95% CI: 0.02–0.23).

**Table 1:** Mortality, incidence and prevalence rates attributed to CNS neoplasms in Middle East and North Africa (MENA) region in 1990.

	1990					
	Incidence		Prevalence		Mortality	
	Cases	Age-adjusted rate*	Cases	Age-adjusted rate*	Cases	Age-adjusted rate*
Overall	15,311.96 *(11,074.86–20,666.80*)	6.04 *(4.33–8.35*)	42,042.43 *(26,440.09–65,287.03*)	13.46 *(8.42–20.80*)	10,856.45 *(8023.82–14,749.90*)	4.80 *(3.49–6.92*)
Gender						
Male	8418.74 *(5572.77–12,711.66*)	6.52 *(4.38–10.34*)	21,915.73 *(12,816.03–35,223.46*)	13.74 *(8.30–22.08*)	6128.46 *(4031.89–9301.93*)	5.30 *(3.57–8.26*)
Female	6893.22 *(4551.13–10,295.76*)	5.53 *(3.71–8.56*)	20,126.70 *(11,116.06–34,330.23*)	13.15 *(7.43–22.52*)	4727.99 *(3223.86–7114.53*)	4.27 *(2.93–6.70*)
Age group†						
<20 yr	5593.15 *(3911.58–7756.28*)	3.14 *(2.20–4.36*)	20,835.21 *(12,245.91–33,264.24*)	11.72 *(6.89–18.70*)	3145.27 *(2194.01–4133.52*)	1.77 *(1.23–2.32*)
20+ yr	9718.81 *(6945.69–13,653.74*)	5.97 *(4.27–8.39*)	21,207.22 *(13,044.54–32,709.77*)	13.03 *(8.01–20.10*)	7711.18 *(5566.70–11,058.28*)	4.74 *(3.42–6.79*)

CNS = central nervous system, MENA = Middle East and North Africa.

* All reported rates are expressed per 1,00,000 population.

† Age-standardized rates are not applicable for analyses stratified by age groups.

**Table 2:** Mortality, incidence and prevalence rates attributed to CNS neoplasms in Middle East and North Africa (MENA) region in 2023.

	2023					
	Incidence		Prevalence		Mortality	
	Cases	Age-adjusted rate*	Cases	Age-adjusted rate*	Cases	Age-adjusted rate*
Overall	38,563.96 *(28,667.46–51,035.62*)	6.94 *(5.18–9.28*)	1,19,222.46 *(71,124.41–1,91,073.97*)	19.51 *(11.71–31.31*)	26,329.42 *(20,624.60–34,267.37*)	5.01 *(3.96–6.61*)
Gender						
Male	20,858.34 *(14,834.08–28,897.18*)	7.36 *(5.22–10.27*)	59,960.30 *(37,902.08–94,093.00*)	19.10 *(12.11–30.18*)	14,782.15 *(10,671.93–20,017.14*)	5.54 *(3.99–7.57*)
Female	17,705.62 *(12,147.16–25,626.13*)	6.52 *(4.50–9.55*)	59,262.16 *(31,866.62–1,03,361.36*)	20.02 *(10.85–34.70*)	11,547.27 *(7762.15–16,237.39*)	4.47 *(3.06–6.31*)
Age group†						
<20 yr	6761.15 *(4758.09–9303.81*)	2.83 *(1.99–3.90*)	32,785.02 *(18,922.04–53,516.75*)	13.73 *(7.92–22.41*)	3157.15 *(2275.85–3965.07*)	1.32 *(0.95–1.66*)
20+ yr	31,802.81 *(23,711.40–42,316.10*)	7.97 *(5.94–10.60*)	86,437.44 *(52,174.22–1,38,557.91*)	21.65 *(13.07–34.71*)	23,172.27 *(18,203.32–30,510.83*)	5.80 *(4.56–7.64*)

CNS = central nervous system, MENA = Middle East and North Africa.

* All reported rates are expressed per 1,00,000 population.

† Age-standardized rates are not applicable for analyses stratified by age groups.

**Table 3:** Mortality, incidence and prevalence rates attributed to CNS neoplasms in Middle East and North Africa (MENA) region from 1990 to 2023.

Variable		Trend Segment	Start point	End point	Annual percentage change (APC)	Average annual percentage change (AAPC)	*P* value
Mortality	Overall	1	1990	1999	0.07 (−0.12 to 0.26)	0.09* (0.02–0.17)	.008867
		2	1999	2016	0.41* (0.34–0.47)		
		3	2016	2023	−0.63* (−0.83 to −0.42)		
	Female	1	1990	1998	0.11 (−0.03 to 0.25)	0.13* (0.02–0.23)	.018219
		2	1998	2013	0.32* (0.26–0.37)		
		3	2013	2016	0.82 (−0.28 to 1.93)		
		4	2016	2023	−0.55* (−0.69 to −0.40)		
	Male	1	1990	1994	0.60* (0.16–1.04)	0.13 (0.00–0.26)	.050581
		2	1994	2000	−0.30* (−0.59 to 0.00)		
		3	2000	2005	0.94* (0.57–1.30)		
		4	2005	2017	0.29* (0.22–0.36)		
		5	2017	2020	−1.30* (−2.30 to −0.30)		
		6	2020	2023	−0.15* (−0.66 to 0.36)		
	<20 yr	1	1990	2001	−1.27* (−1.38 to −1.16)	−0.90* (−1.10 to −0.71)	<.000001
		2	2001	2015	−0.57* (−0.65 to −0.50)		
		3	2015	2018	−1.80* (−3.17 to −0.40)		
		4	2018	2021	0.91 (−0.48 to 2.32)		
		5	2021	2023	−2.50* (−3.97 to −1.02)		
	20+ yr	1	1990	2000	0.09 (−0.05 to 0.24)	0.58* (0.50–0.67)	<.000001
		2	2000	2010	0.54* (0.40–0.68)		
		3	2010	2017	1.40* (1.16–1.64)		
		4	2017	2023	0.51* (0.28–0.75)		
Incidence	Overall	1	1990	1993	0.71* (0.14–1.28)	0.42* (0.30–0.54)	<.000001
		2	1993	2000	−0.05 (−0.24 to 0.15)		
		3	2000	2003	1.17* (0.14–2.20)		
		4	2003	2010	0.45* (0.29–0.61)		
		5	2010	2016	0.68* (0.48–0.89)		
		6	2016	2023	0.20* (0.07–0.33)		
	Female	1	1990	2000	0.23* (0.14–0.32)	0.49* (0.38–0.59)	<.000001
		2	2000	2003	0.88 (−0.14 to 1.90)		
		3	2003	2011	0.40* (0.27–0.54)		
		4	2011	2016	0.99* (0.66–1.31)		
		5	2016	2023	0.42* (0.29–0.56)		
	Male	1	1990	1994	0.64* (0.12–1.16)	0.36* (0.25–0.47)	<.000001
		2	1994	2000	−0.25 (−0.58 to 0.09)		
		3	2000	2005	1.08* (0.65–1.50)		
		4	2005	2016	0.47* (0.38–0.57)		
		5	2016	2023	0.05 (−0.12 to 0.21)		
	<20 yr	1	1990	2001	−1.03* (−1.12 to −0.94)	−0.35* (−0.52 to −0.17)	.000101
		2	2001	2015	−0.03 (−0.10 to 0.04)		
		3	2015	2018	−0.78 (−2.05 to 0.51)		
		4	2018	2021	1.90* (0.62–3.20)		
		5	2021	2023	−1.47* (−2.70 to −0.22)		
	20+ yr	1	1990	1993	0.74* (0.13–1.36)	0.88* (0.76–1.00)	<.000001
		2	1993	2000	0.07 (−0.13 to 0.27)		
		3	2000	2003	1.27* (0.22–2.34)		
		4	2003	2010	0.61* (0.45–0.77)		
		5	2010	2017	1.61* (1.45–1.77)		
		6	2017	2023	1.17* (0.99–1.34)		
Prevalence	Overall	1	1990	1993	1.16* (0.44–1.88)	1.13* (1.03–1.23)	<.000001
		2	1993	1998	0.06 (−0.40 to 0.53)		
		3	1998	2015	1.14* (1.08–1.19)		
		4	2015	2023	1.77* (1.61–1.94)		
	Female	1	1990	1993	1.22* (0.70–1.75)	1.30* (1.21–1.40)	<.000001
		2	1993	1997	0.17 (−0.35 to 0.68)		
		3	1997	2013	1.14* (1.10–1.19)		
		4	2013	2020	1.75* (1.58–1.93)		
		5	2020	2023	2.74* (2.20–3.27)		
	Male	1	1990	1994	0.93* (0.40–1.45)	1.01* (0.89–1.14)	<.000001
		2	1994	1998	−0.39 (−1.18 to 0.41)		
		3	1998	2018	1.14* (1.10–1.19)		
		4	2018	2023	1.70* (1.36–2.04)		
	<20 yr	1	1990	1994	−0.12 (−0.52 to 0.29)	0.47* (0.27–0.66)	.000002
		2	1994	1998	−1.35* (−2.01 to −0.69)		
		3	1998	2018	0.65* (0.61–0.70)		
		4	2018	2021	2.86* (1.17–4.57)		
		5	2021	2023	−0.06 (−1.72 to 1.62)		
	20+ yr	1	1990	1999	0.76* (0.55–0.98)	1.52* (1.44–1.60)	<.000001
		2	1999	2013	1.45* (1.35–1.56)		
		3	2013	2023	2.31* (2.18–2.44)		

AAPC = average annual percent change, APC = annual percent change, CNS = central nervous system, MENA = Middle East and North Africa.

* Indicates that the APC is significantly different from 0 at the alpha = 0.05 level.

**Figure 1.**
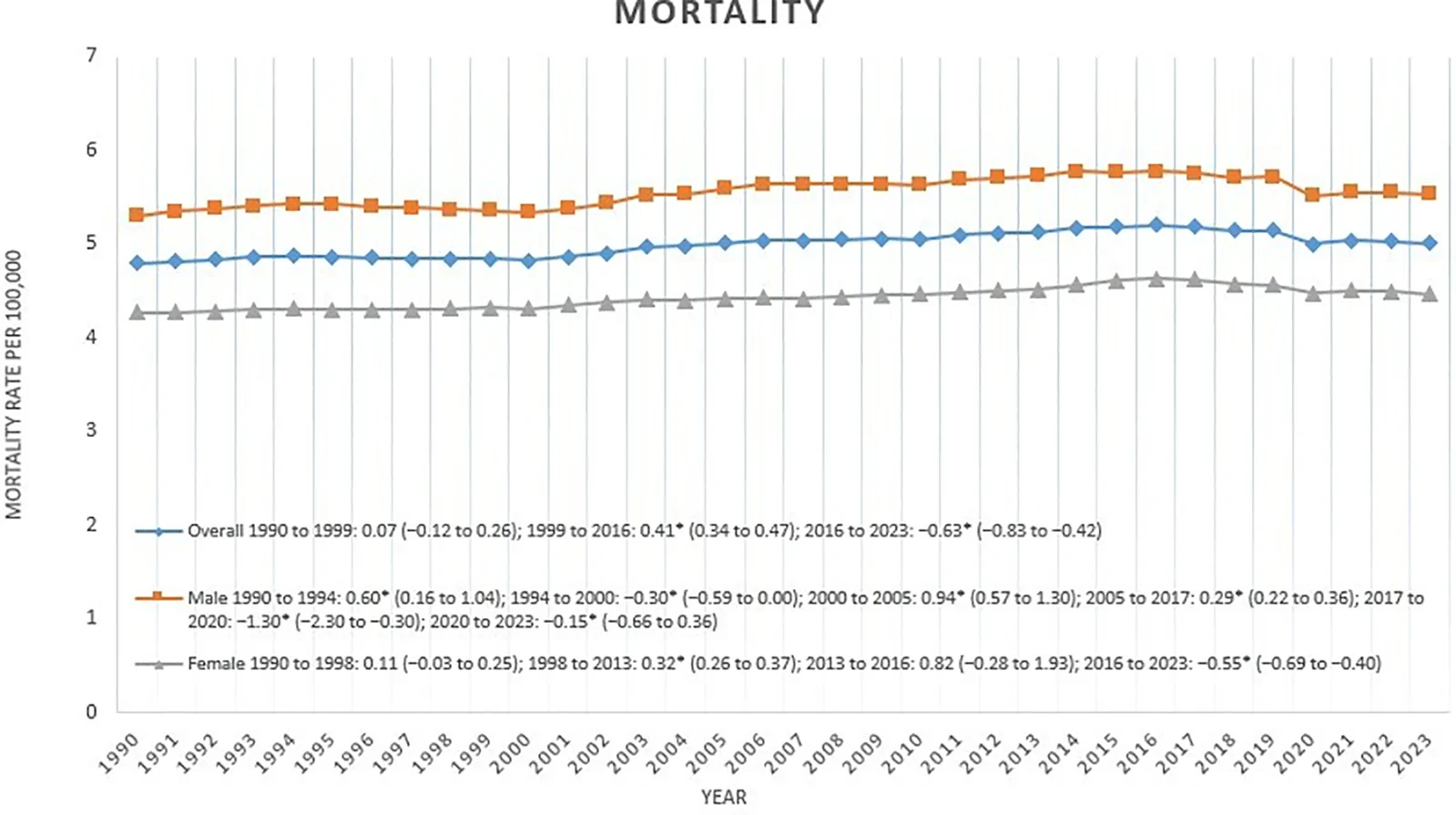
Mortality rate changes from 1990 to 2023 in MENA countries stratified by sex. MENA = Middle East and North Africa.

Based on overall total change in CNS neoplasm-related mortality, Egypt demonstrated the greatest increase over the study period, with a total change of 0.66 (95% CI: −0.16 to 2.05). Iraq showed the second largest increase, with a total change of 0.26 (95% CI: −0.05 to 0.88). Conversely, the largest declines were observed in the United Arab Emirates and Qatar. The United Arab Emirates experienced the greatest decrease, with a total change of −0.53 (95% CI: −0.68 to −0.32), followed by Qatar, which showed a marked reduction of −0.48 (95% CI: −0.64 to −0.27; Table S1, Supplemental Digital Content 8, Fig. S3, Supplemental Digital Content 6).

From 2023 to 2050, ASMR is projected to increase by approximately 33.8%. This increase is expected to be more pronounced among females, with an estimated increase of 35.8%, compared with a 31.8% increase among males (Figs. S4–S6, Supplemental Digital Content 3).

#### 3.1.2. Age groups

Overall, mortality trends differed by age group, with younger individuals experiencing declining age-standardized rates, while adults showed a sustained increase in age-adjusted mortality over the study period. Among individuals younger than 20 years, the number of deaths remained largely stable, increasing slightly from 3145.27 (95% CI: 2194.01–4133.52) in 1990 to 3157.15 (95% CI: 2275.85–3965.07) in 2023. In contrast, age-standardized mortality declined over the same period, with the ASMR decreasing from 1.77 (95% CI: 1.23–2.32) per 1,00,000 in 1990 to 1.32 (95% CI: 0.95–1.66) per 1,00,000 in 2023, corresponding to a significant downward trend (AAPC − 0.90%*, 95% CI: −1.10 to −0.71). Among adults aged 20 years and older, total deaths increased substantially, rising from 7711.18 (95% CI: 5566.70–11,058.28) in 1990 to 23,172.27 (95% CI: 18,203.32–30,510.83) in 2023. The ASMR in this age group increased from 4.74 (95% CI: 3.42–6.79) per 1,00,000 in 1990 to 5.80 (95% CI: 4.56–7.64) per 1,00,000 in 2023, with a statistically significant upward trend (AAPC 0.58%*, 95% CI: 0.50–0.67; Fig. 2).

**Figure 2.**
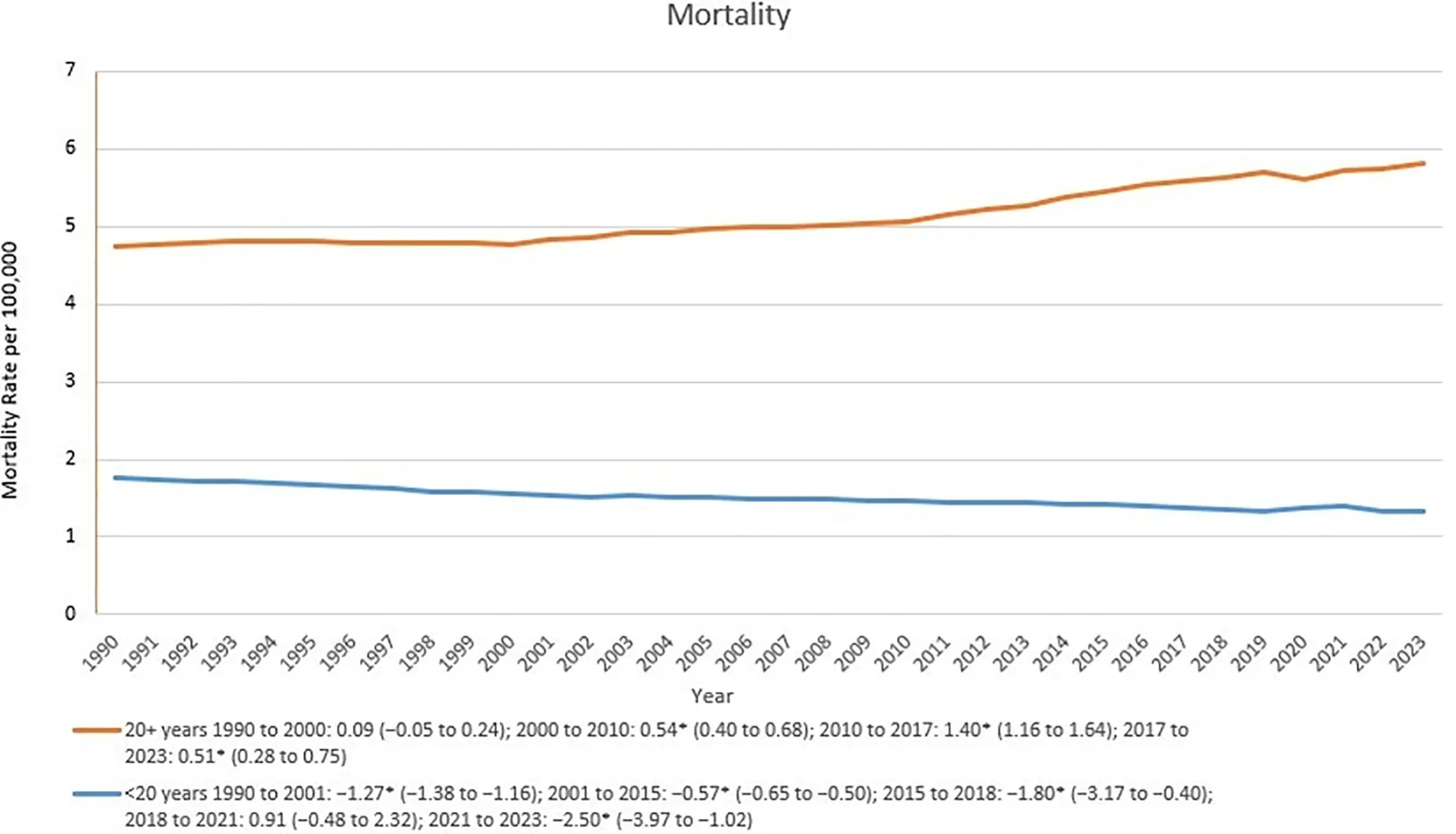
Mortality rate changes from 1990 to 2023 in MENA countries stratified by age groups. MENA = Middle East and North Africa.

### 3.2. Incidence

#### 3.2.1. Overall and gender

Between 1990 and 2023, the incidence of CNS neoplasms increased markedly in absolute numbers and also rose after adjustment for age. A total of 8,50,977 new cases were recorded over the study period. Annual incident cases increased from 15,311.96 (95% CI: 11,074.86–20,666.80) in 1990 to 38,563.96 (95% CI: 28,667.46–51,035.62) in 2023. The age-standardized incidence rate (ASIR) increased from 6.04 (95% CI: 4.33–8.35) per 1,00,000 in 1990 to 6.94 (95% CI: 5.18–9.28) per 1,00,000 in 2023, corresponding to a statistically significant upward trend (AAPC 0.42%*, 95% CI: 0.30–0.54; Tables 1, 2 and Figs. S7, S8, Supplemental Digital Content 1). By sex, incidence rates were consistently higher among males. The male ASIR rose from 6.52 (95% CI: 4.38–10.34) in 1990 to 7.36 (95% CI: 5.22–10.27) in 2023, with a significant increase over time (AAPC 0.36%*; 95% CI: 0.25–0.47). Females demonstrated a comparable rise, with ASIR increasing from 5.53 (95% CI: 3.71–8.56) to 6.52 (95% CI: 4.50–9.55), with an AAPC of 0.49%* (95% CI: 0.38–0.59; Table 3, Fig. 3).

**Figure 3.**
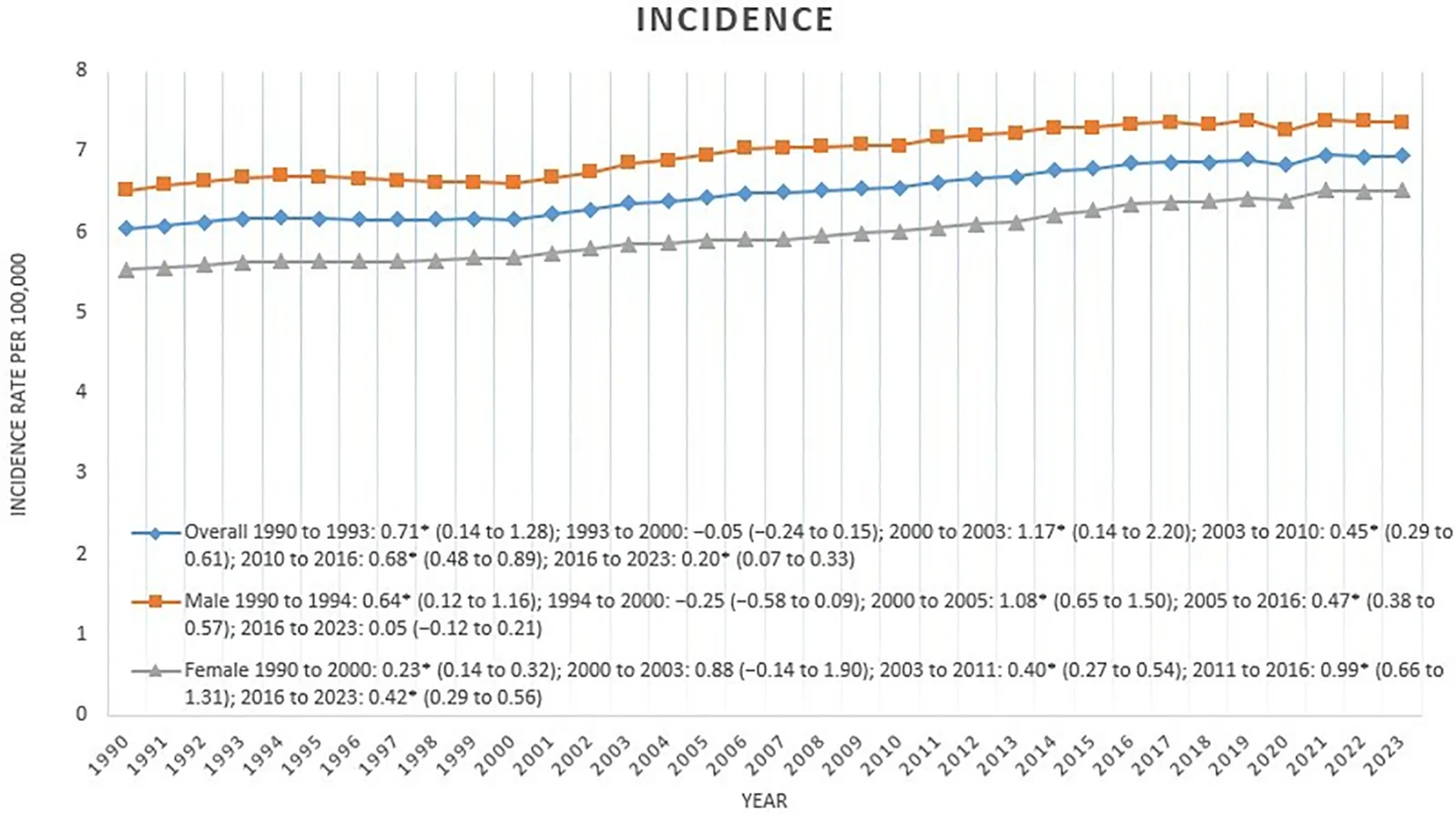
Incidence rate changes from 1990 to 2023 in MENA countries stratified by sex. MENA = Middle East and North Africa.

Between 1990 and 2023, the incidence of CNS neoplasms varied across countries in the MENA region. The most pronounced increases were observed in Egypt and Iraq. In Egypt, the overall incidence rose by 0.72 (95% CI: −0.11 to 2.00). Iraq followed, with an increase of 0.40 (95% CI: 0.00–1.01). In contrast, the steepest declines were observed in the United Arab Emirates and Kuwait. The incidence in the UAE decreased by −0.50 (95% CI: −0.68 to −0.30), while Kuwait showed a reduction of −0.35 (95% CI: −0.55 to −0.13; Table S1, Supplemental Digital Content 8, Fig. S9, Supplemental Digital Content 7).

#### 3.2.2. Age groups

Among individuals younger than 20 years, the number of new cases of CNS neoplasms increased modestly from 5593.15 (95% CI: 3911.58–7756.28) in 1990 to 6761.15 (95% CI: 4758.09–9303.81) in 2023. In contrast, the ASIR declined slightly from 3.14 (95% CI: 2.20–4.36) to 2.83 (95% CI: 1.99–3.90), corresponding to a significant downward trend (AAPC −0.35%*, 95% CI: −0.52 to −0.17). Among adults aged 20 years and older, incident cases rose substantially, from 9718.81 (95% CI: 6945.69–13,653.74) in 1990 to 31,802.81 (95% CI: 23,711.40–42,316.10) in 2023. The ASIR increased from 5.97 (95% CI: 4.27–8.39) per 1,00,000 in 1990 to 7.97 (95% CI: 5.94–10.60) in 2023, with a significant upward trend (AAPC 0.88%*, 95% CI: 0.76–1.00; Fig. 4).

**Figure 4.**
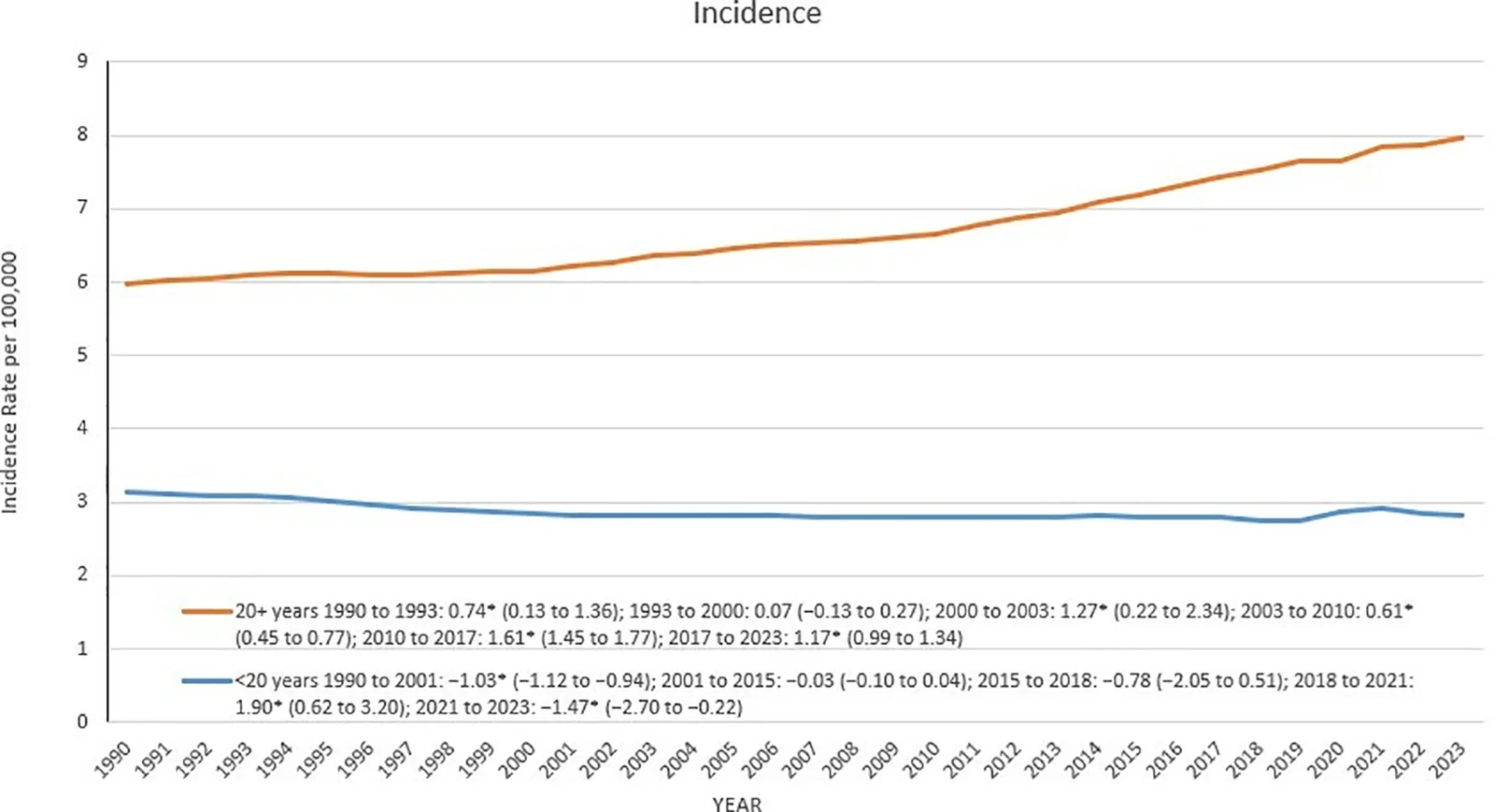
Incidence rate changes from 1990 to 2023 in MENA countries stratified by age groups. MENA = Middle East and North Africa.

### 3.3. Prevalence

#### 3.3.1. Overall and gender

From 1990 to 2023, the prevalence of CNS neoplasms in the MENA region increased substantially in absolute numbers as well as after adjusting for age. Total prevalent cases rose from 42,042.43 (95% CI: 26,440.09–65,287.03) in 1990 to 1,19,222.46 (95% CI: 71,124.41–1,91,073.97) in 2023. The age-standardized prevalence rate (ASPR) increased from 13.46 (95% CI: 8.42–20.80) per 1,00,000 in 1990 to 19.51 (95% CI: 11.71–31.31) per 1,00,000 in 2023, corresponding to a significant upward trend (AAPC 1.13%*, 95% CI: 1.03–1.23; Tables 1, 2 and Figs. S10, S11, Supplemental Digital Content 2). By sex, males showed an increase in ASPR from 13.74 (95% CI: 8.30–22.08) to 19.10 (95% CI: 12.11–30.18), with an AAPC of 1.01%* (95% CI: 0.89–1.14). Females experienced a slightly higher rise, from 13.15 (95% CI: 7.43–22.52) to 20.02 (95% CI: 10.85–34.70), corresponding to an AAPC of 1.30%* (95% CI: 1.21–1.40; Table 3, Fig. 5). Overall, prevalence of CNS neoplasms has increased steadily over the past 3 decades, with age-standardized rates rising more prominently among females.

**Figure 5.**
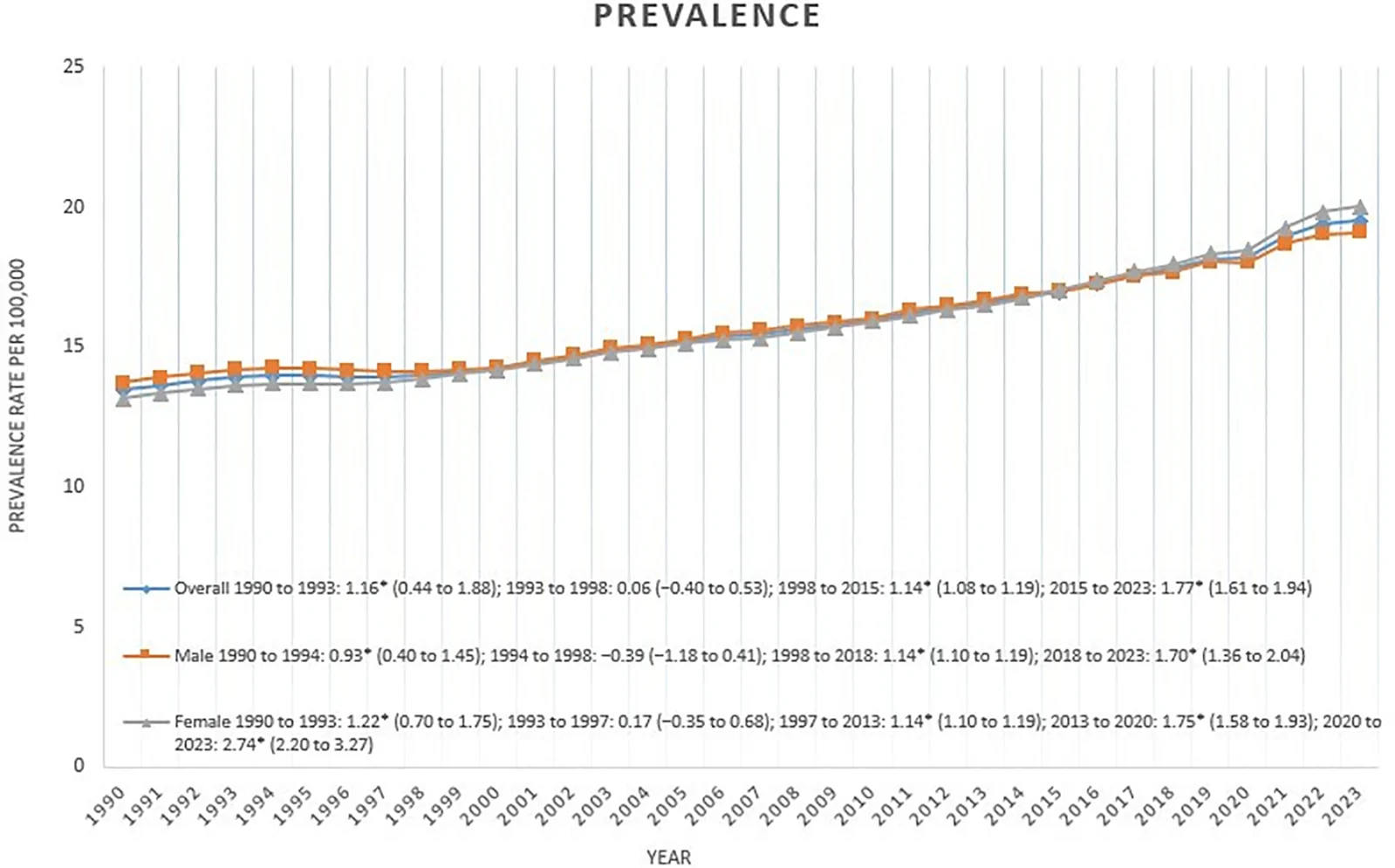
Prevalence rate changes from 1990 to 2023 in MENA countries stratified by sex. MENA = Middle East and North Africa.

The largest increases were observed in Egypt and Lebanon. In Egypt, the overall prevalence rose by 0.89 (95% CI: −0.07 to 2.78), while Lebanon showed an increase of 1.06 (95% CI: 0.03–3.06). In contrast, the greatest declines were observed in the United Arab Emirates and Kuwait, with reductions of −0.35 (95% CI: −0.68 to 0.19) and −0.12 (95% CI: −0.60 to 0.80), respectively (Table S1, Supplemental Digital Content 8, Fig. S12, Supplemental Digital Content 5).

#### 3.3.2. Age groups

Between 1990 and 2023, the prevalence of CNS neoplasms in the MENA region showed marked increases across all age groups. Among individuals younger than 20 years, prevalent cases rose from 20,835.21 (95% CI: 12,245.91–33,264.24) in 1990 to 32,785.02 (95% CI: 18,922.04–53,516.75) in 2023. The corresponding ASPR increased from 11.72 (95% CI: 6.89–18.70) to 13.73 (95% CI: 7.92–22.41), reflecting a significant upward trend (AAPC 0.47%*, 95% CI: 0.27–0.66; Fig. 6). Among adults aged 20 years and older, prevalent cases rose sharply from 21,207.22 (95% CI: 13,044.54–32,709.77) in 1990 to 86,437.44 (95% CI: 52,174.22–1,38,557.91) in 2023. The ASPR increased from 13.03 (95% CI: 8.01–20.10) to 21.65 (95% CI: 13.07–34.71), with a significant upward trend (AAPC 1.52%*, 95% CI: 1.44–1.60).

**Figure 6.**
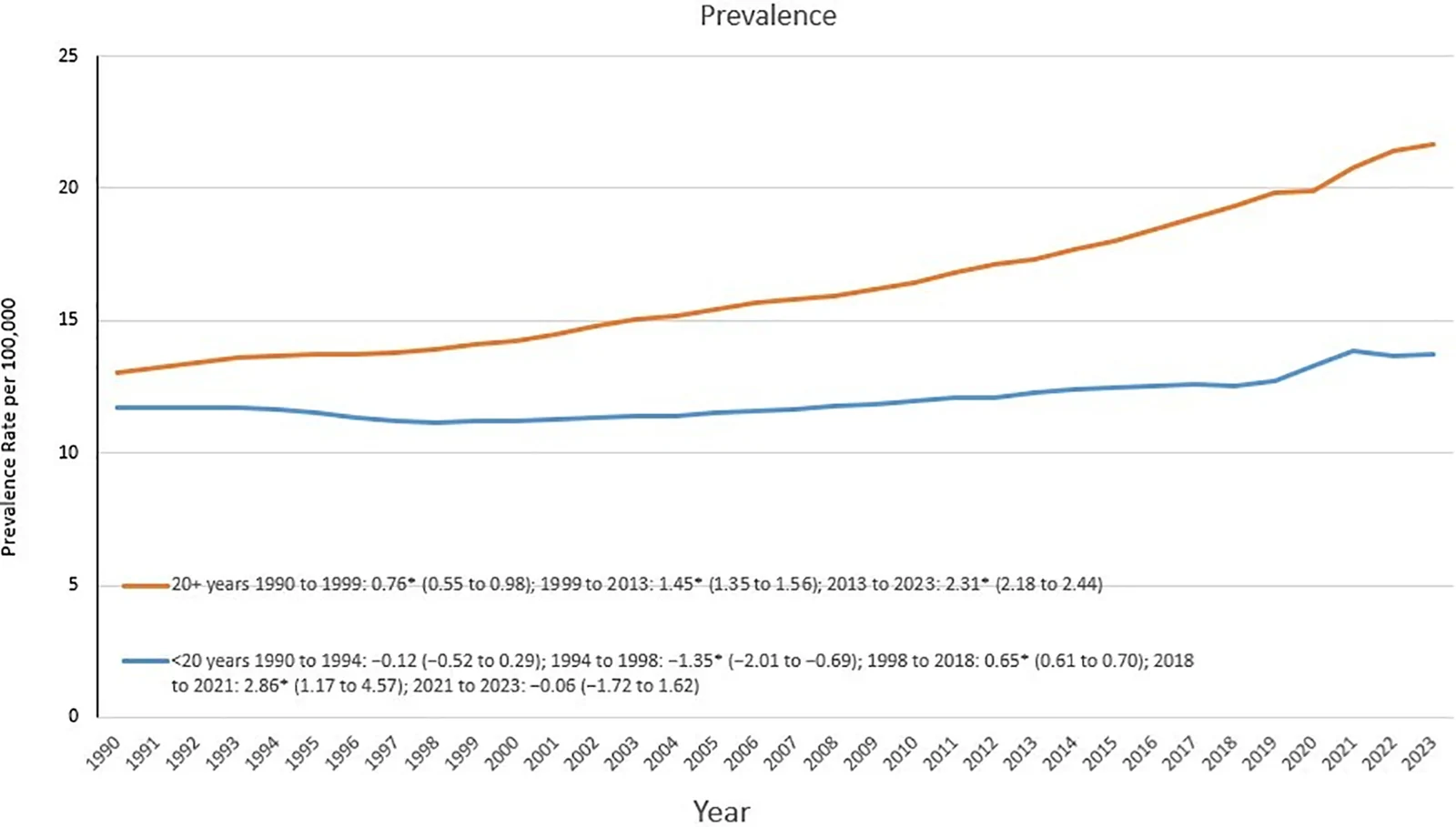
Prevalence rate changes from 1990 to 2023 in MENA countries stratified by age groups. MENA = Middle East and North Africa.

## 4. Discussion

Our analysis of long-term trends in CNS neoplasm burden across the MENA region revealed distinct patterns in mortality, incidence, and prevalence from 1990 to 2023. Overall mortality increased in absolute numbers, while age-standardized rates remained largely stable. Males consistently exhibited higher rates than females, though females showed a significant upward trend over time. Age-stratified analysis highlighted divergent patterns: younger individuals experienced declining ASMR, whereas adults showed sustained increases. Incidence mirrored these patterns, rising in both absolute and age-adjusted terms, with males experiencing higher rates but females demonstrating slightly faster proportional growth. Country-level differences were notable, with Egypt and Iraq showing the largest increases in both mortality and incidence, while the United Arab Emirates, Qatar, and Kuwait experienced declines. Prevalence rose steadily across the region, with ASPRs increasing more prominently among females. ASPR also revealed greater increases among adults compared with younger populations. Together, these findings suggest that CNS neoplasm burden in the MENA region has intensified over the past 3 decades, shaped by demographic shifts, sex-specific vulnerabilities, and regional variations in risk exposures and healthcare access.

Our findings are consistent with previous large database studies highlighting the increasing burden of CNS neoplasms in MENA countries.^[12,19]^ The development of CNS cancers is complex and reflects the interplay of genetic, environmental, lifestyle, and immune influences. Genetic susceptibility, particularly inherited and acquired gene mutations, plays a central role. Familial cancer syndromes such as Li–Fraumeni syndrome, neurofibromatosis, and retinoblastoma are associated with a markedly higher risk of CNS tumors. These conditions are commonly linked to defects in tumor suppressor genes that disrupt normal cellular growth control.^[20,21]^ High-income countries, including several in the Middle East, have more advanced genetic screening, improved surveillance, and mutation detection systems. This allows earlier recognition of high-risk individuals and may help explain the relatively higher burden observed in these regions.^[22]^

Glioblastomas, the most frequent malignant brain tumors, are diagnosed about twice as often in males and are associated with more severe cognitive impairment compared to females. Survival outcomes are also poorer among male patients. Experimental evidence suggests that the retinoblastoma protein, a key regulator that suppresses tumor development, shows lower activity in male brain cells than in female brain cells, which may partly explain these sex-based differences.^[23]^ Evidence also indicates that estrogen and progesterone may have neuroprotective roles and can modulate tumor development and progression.^[24,25]^ In addition, occupational exposures represent an important contributor, especially in male-dominated occupations where workers are more frequently exposed to potential carcinogens, including pesticides, ionizing radiation, and electromagnetic fields.^[26]^

Our analysis demonstrated an increasing burden of CNS neoplasms among adults, alongside a decline in burden within younger age groups. Recent improvements in prenatal diagnostic methods and screening for tumor-predisposing syndromes have likely played a role in reducing the incidence of CNS neoplasms among younger populations.^[27]^ Consistent with previous research, our results also indicate a steady decline in mortality from childhood brain and CNS cancers.^[28]^ This improvement is likely driven by standardized treatment protocols, more precise grading of therapy intensity, and advances in treatments, including tumor vaccines, stem cell therapy, and oncolytic viruses.^[29,30]^ Lifestyle factors – including obesity, physical inactivity, and dietary shifts – may increase the risk of CNS cancers in adults, especially in high-income regions of the Middle East experiencing rapid industrialization and urbanization.^[31]^

Country-level analysis revealed mixed trends, with nations such as Egypt and Iraq experiencing a higher burden than others. The underlying causes of these variations are not fully understood but may involve changes in exposure to risk factors, broader genetic screening for high-risk germline mutations, and advances in treatment. Nevertheless, these trends should be interpreted cautiously, as CNS cancers encompass diverse histological subtypes, and shifts in diagnostic practices and registry criteria over time can influence reported patterns.^[32]^ In some cases, comparisons between countries are further complicated by diagnostic challenges, inconsistent definitions, and evolving classification standards.^[33,34]^ Environmental and occupational hazards – including pesticide use, ionizing radiation, and potential war-related toxins – are frequently cited as contributing factors, yet definitive population-level evidence linking them to cancer incidence in Egypt and Iraq remains scarce.^[35-37]^ Unequal access to essential diagnostics and oncologic services (such as advanced neuroimaging, pathology, and specialized neurosurgical or radiation care) further skews reported patterns of disease burden and survival.^[38]^ Compounding these disparities, variations in registry infrastructure – which predominantly relies on hospital records rather than comprehensive, population-based tracking – make cross-country comparisons difficult. Broader demographic dynamics and underlying genetic predispositions may also play a role, though their precise impact remains largely uncharacterized.

Several limitations of GBD-based analyses should be considered. First, all estimates rely on the Global Burden of Disease modeling framework, which synthesizes data from cancer registries, vital registration systems, and verbal autopsy sources of uneven completeness and quality. In several Middle Eastern and North African countries, particularly those affected by conflict or limited surveillance capacity, sparse primary data require heavier reliance on statistical modeling, increasing uncertainty despite the reporting of uncertainty intervals. Unequal registry coverage and differences in diagnostic and coding practices may introduce coverage, information, and misclassification bias. Temporal improvements in neuroimaging, diagnostic capacity, and cancer registration may also introduce ascertainment bias, such that increasing incidence and prevalence partly reflect improved detection rather than a true increase in disease occurrence. Conversely, declining trends in some Gulf countries may be influenced by population dynamics, expatriate demographics, or registry coverage rather than reduced risk. Also, the GBD framework does not allow further economic or healthcare-system stratification of MENA countries. Second, CNS neoplasms are analyzed as a single aggregated category. The GBD framework does not allow stratification by histology, tumor grade, molecular characteristics, or anatomical site. Given the marked heterogeneity in etiology, prognosis, and treatment response across CNS tumor subtypes, aggregated estimates may obscure clinically meaningful differences in burden and temporal trends. As this was an ecological analysis, ecological bias and residual confounding cannot be excluded, and associations with healthcare access, environmental exposures, lifestyle, or socioeconomic factors should not be interpreted as individual-level causal relationships. Finally, age standardization supports comparisons but cannot fully account for migration, demographic transitions, or unequal access to specialized neuro-oncological care. Projections to 2050 depend on assumptions about demographic change and historical trends and should therefore be interpreted as indicative rather than predictive.

## 5. Conclusion

Despite significant advances in medical technology and therapeutic approaches, CNS neoplasms continue to pose substantial clinical challenges due to their complexity, aggressive behavior, and intricate treatment requirements. Early detection remains essential for improving survival outcomes, yet the long-term consequences – including cognitive deficits, neurological impairments, and physical disabilities – impose a profound burden on patients, caregivers, and healthcare infrastructures. Our analysis indicates that the burden of CNS neoplasms in the MENA region has intensified over the past 3 decades. While younger populations have experienced some reductions in incidence and mortality, adults, particularly in countries such as Egypt and Iraq, have faced rising trends in both incidence and mortality. Overall, CNS neoplasms represent an increasingly important contributor to neurological and oncological burden in the region. Strengthening population-based cancer surveillance, improving diagnostic accuracy, and expanding access to specialized neuro-oncological services are critical, particularly in countries with rapidly rising burden. These findings also underscore the need for healthcare professionals to intensify tailored efforts in prevention through lifestyle modification targeting known risk factors, implement context-specific screening programs, and provide structured management to mitigate the CNS cancer burden. Future research incorporating subtype-specific data, survival outcomes, and health system indicators will be essential to guide prevention strategies, resource allocation, and long-term policy planning in this region.

## Author contributions

**Conceptualization:** Ibraheem Altamimi, Abdullah Abdulaziz Alnaim.

**Data curation:** Ibraheem Altamimi.

**Formal analysis:** Abdullah Abdulaziz Alnaim.

**Investigation:** Raghad Emad Alhalabi.

**Methodology:** Yousef Saeed Alzahrani, Raghad Emad Alhalabi.

**Project administration:** Ibraheem Altamimi, Ahmed Khalid Alzahrani, Norah Abdullah Alodhaib, Jaber Abdullah Alshahrani.

**Software:** Ibraheem Altamimi.

**Supervision:** Ibraheem Altamimi, Ahmed Khalid Alzahrani, Norah Abdullah Alodhaib.

**Visualization:** Abdulelah Mohammed Abdullah Alsakti, Norah Abdullah Alodhaib.

**Validation:** Ali Abdulhakim Alhakeem.

**Writing – original draft:** Ibraheem Altamimi, Alaa Alanazi, Abdullah Abdulaziz Alnaim, Ahmed Khalid Alzahrani, Yousef Saeed Alzahrani, Abdulelah Mohammed Abdullah Alsakti, Ali Abdulhakim Alhakeem, Mohammed Mastour Alharthi, Raghad Emad Alhalabi, Aalaa Saleh, Jaber Abdullah Alshahrani.

**Writing – review & editing:** Ibraheem Altamimi, Alaa Alanazi, Abdullah Abdulaziz Alnaim, Ahmed Khalid Alzahrani, Yousef Saeed Alzahrani, Abdulelah Mohammed Abdullah Alsakti, Ali Abdulhakim Alhakeem, Mohammed Mastour Alharthi, Raghad Emad Alhalabi, Aalaa Saleh, Jaber Abdullah Alshahrani.
















