# Supplementary material for: Burden of CNS neoplasms in the Middle East and North Africa from 1990 to 2023, with projections to 2050: Insights from Global Burden of Disease
Source: Medicine (Baltimore). 2026 Oct 16;105(42):e51128. doi: 10.1097/MD.0000000000051128 (PMC13681167; doi:10.1097/MD.0000000000051128)
Supplement: Supplementary file 1 [file medi-105-e51128-s008.docx]

**Supplementary Table 1** Age standardized incidence (ASIR), Prevalence (ASPR) and mortality (ASMR) changes across MENA countries from 1990 to 2023

| **Measure** | **Country** | **Sex** | **Rate of Change** | **Upper Confidence Interval** | **Lower Confidence Interval** |
| --- | --- | --- | --- | --- | --- |
| Deaths | Palestine | Male | -0.08965 | 0.402581 | -0.40621 |
| Deaths | Palestine | Female | -0.03419 | 0.472009 | -0.40851 |
| Deaths | Palestine | Both | -0.06212 | 0.289703 | -0.35678 |
| Deaths | Oman | Male | -0.01452 | 0.694324 | -0.3732 |
| Deaths | Oman | Female | -0.06297 | 0.575784 | -0.41473 |
| Deaths | Oman | Both | -0.04213 | 0.370104 | -0.29089 |
| Deaths | Qatar | Male | -0.50036 | -0.18587 | -0.68885 |
| Deaths | Qatar | Female | -0.47375 | -0.16304 | -0.66774 |
| Deaths | Qatar | Both | -0.48272 | -0.27063 | -0.63754 |
| Deaths | Saudi Arabia | Male | -0.0658 | 0.438812 | -0.41847 |
| Deaths | Saudi Arabia | Female | 0.199845 | 0.736899 | -0.29508 |
| Deaths | Saudi Arabia | Both | 0.021236 | 0.437502 | -0.32689 |
| Deaths | Sudan | Male | 0.146314 | 0.861441 | -0.28305 |
| Deaths | Sudan | Female | 0.165018 | 0.969921 | -0.27333 |
| Deaths | Sudan | Both | 0.152781 | 0.642772 | -0.15327 |
| Deaths | Syrian Arab Republic | Male | -0.1151 | 0.533726 | -0.47111 |
| Deaths | Syrian Arab Republic | Female | -0.07567 | 0.45003 | -0.46049 |
| Deaths | Syrian Arab Republic | Both | -0.09842 | 0.377747 | -0.36622 |
| Deaths | Egypt | Male | 0.572614 | 1.834923 | -0.19705 |
| Deaths | Egypt | Female | 0.76647 | 3.133615 | -0.24551 |
| Deaths | Egypt | Both | 0.662571 | 2.054031 | -0.16468 |
| Deaths | Tunisia | Male | -0.33165 | 0.070813 | -0.55076 |
| Deaths | Tunisia | Female | -0.36414 | 0.04584 | -0.5792 |
| Deaths | Tunisia | Both | -0.34829 | -0.09183 | -0.50794 |
| Deaths | Iran (Islamic Republic of) | Male | -0.02072 | 0.583287 | -0.36022 |
| Deaths | Iran (Islamic Republic of) | Female | -0.11996 | 0.453243 | -0.46607 |
| Deaths | Iran (Islamic Republic of) | Both | -0.07072 | 0.387205 | -0.31139 |
| Deaths | Iraq | Male | 0.234208 | 1.09674 | -0.19448 |
| Deaths | Iraq | Female | 0.297099 | 1.385835 | -0.1957 |
| Deaths | Iraq | Both | 0.261542 | 0.881968 | -0.05347 |
| Deaths | TÃ¼rkiye | Male | 0.050409 | 0.672105 | -0.27675 |
| Deaths | TÃ¼rkiye | Female | -0.07094 | 0.400163 | -0.38639 |
| Deaths | TÃ¼rkiye | Both | -0.00937 | 0.395816 | -0.26567 |
| Deaths | Afghanistan | Male | 0.038591 | 0.678973 | -0.34379 |
| Deaths | Afghanistan | Female | 0.116868 | 0.81396 | -0.32092 |
| Deaths | Afghanistan | Both | 0.074697 | 0.489421 | -0.19492 |
| Deaths | United Arab Emirates | Male | -0.593 | -0.28967 | -0.75231 |
| Deaths | United Arab Emirates | Female | -0.39241 | 0.001313 | -0.64389 |
| Deaths | United Arab Emirates | Both | -0.53049 | -0.31504 | -0.68415 |
| Deaths | Algeria | Male | 0.015992 | 0.488141 | -0.37021 |
| Deaths | Algeria | Female | 0.011542 | 0.604907 | -0.40933 |
| Deaths | Algeria | Both | 0.014799 | 0.322093 | -0.26672 |
| Deaths | Yemen | Male | 0.031306 | 0.638185 | -0.37131 |
| Deaths | Yemen | Female | 0.125347 | 0.857193 | -0.29708 |
| Deaths | Yemen | Both | 0.068828 | 0.476205 | -0.21772 |
| Deaths | Bahrain | Male | -0.22657 | 0.169052 | -0.49124 |
| Deaths | Bahrain | Female | -0.2876 | 0.022747 | -0.57182 |
| Deaths | Bahrain | Both | -0.25125 | 0.010686 | -0.45774 |
| Deaths | Jordan | Male | -0.27146 | 0.157289 | -0.53555 |
| Deaths | Jordan | Female | -0.37277 | 0.001555 | -0.60883 |
| Deaths | Jordan | Both | -0.31504 | -0.01548 | -0.51739 |
| Deaths | Kuwait | Male | -0.42265 | -0.27719 | -0.55066 |
| Deaths | Kuwait | Female | -0.48351 | -0.3548 | -0.60029 |
| Deaths | Kuwait | Both | -0.45005 | -0.36132 | -0.55161 |
| Deaths | Lebanon | Male | 0.201606 | 1.309238 | -0.22341 |
| Deaths | Lebanon | Female | 0.088851 | 0.897713 | -0.29659 |
| Deaths | Lebanon | Both | 0.136465 | 0.808281 | -0.19244 |
| Deaths | Libya | Male | -0.1632 | 0.310856 | -0.47874 |
| Deaths | Libya | Female | -0.14449 | 0.454629 | -0.50573 |
| Deaths | Libya | Both | -0.16232 | 0.141887 | -0.41521 |
| Deaths | Morocco | Male | -0.03957 | 0.494601 | -0.39153 |
| Deaths | Morocco | Female | -0.13592 | 0.374367 | -0.47099 |
| Deaths | Morocco | Both | -0.08549 | 0.195224 | -0.3411 |
| Prevalence | Oman | Male | 0.244027 | 1.378846 | -0.38099 |
| Prevalence | Oman | Female | 0.311601 | 2.032368 | -0.41144 |
| Prevalence | Oman | Both | 0.262662 | 1.261174 | -0.34846 |
| Prevalence | Jordan | Male | -0.0096 | 0.796311 | -0.48876 |
| Prevalence | Jordan | Female | -0.10521 | 1.037372 | -0.60491 |
| Prevalence | Jordan | Both | -0.06066 | 0.799879 | -0.535 |
| Prevalence | United Arab Emirates | Male | -0.3211 | 0.363434 | -0.65503 |
| Prevalence | United Arab Emirates | Female | -0.31885 | 0.599944 | -0.69279 |
| Prevalence | United Arab Emirates | Both | -0.34757 | 0.18815 | -0.6757 |
| Prevalence | Yemen | Male | 0.130389 | 0.826258 | -0.35093 |
| Prevalence | Yemen | Female | 0.366821 | 1.554278 | -0.2902 |
| Prevalence | Yemen | Both | 0.235636 | 0.997341 | -0.27837 |
| Prevalence | Qatar | Male | 0.084648 | 1.124103 | -0.5079 |
| Prevalence | Qatar | Female | 0.09772 | 1.591105 | -0.53261 |
| Prevalence | Qatar | Both | 0.09889 | 1.108409 | -0.50989 |
| Prevalence | Kuwait | Male | -0.15975 | 0.646352 | -0.55431 |
| Prevalence | Kuwait | Female | -0.06115 | 1.27863 | -0.62811 |
| Prevalence | Kuwait | Both | -0.12113 | 0.800396 | -0.59691 |
| Prevalence | Iran (Islamic Republic of) | Male | 0.480008 | 1.937659 | -0.27335 |
| Prevalence | Iran (Islamic Republic of) | Female | 0.443362 | 2.604142 | -0.35811 |
| Prevalence | Iran (Islamic Republic of) | Both | 0.460785 | 1.927375 | -0.29252 |
| Prevalence | Afghanistan | Male | 0.167306 | 0.938236 | -0.33206 |
| Prevalence | Afghanistan | Female | 0.323125 | 1.373175 | -0.23867 |
| Prevalence | Afghanistan | Both | 0.240673 | 0.910861 | -0.23527 |
| Prevalence | Lebanon | Male | 1.107696 | 3.08138 | 0.104381 |
| Prevalence | Lebanon | Female | 1.01764 | 3.675863 | -0.10374 |
| Prevalence | Lebanon | Both | 1.057118 | 3.061897 | 0.029514 |
| Prevalence | Iraq | Male | 0.608854 | 2.409981 | -0.11258 |
| Prevalence | Iraq | Female | 0.874663 | 2.967652 | -0.15118 |
| Prevalence | Iraq | Both | 0.739307 | 2.287964 | -0.07014 |
| Prevalence | Algeria | Male | 0.239689 | 1.366187 | -0.39314 |
| Prevalence | Algeria | Female | 0.369911 | 2.309708 | -0.40502 |
| Prevalence | Algeria | Both | 0.300541 | 1.568096 | -0.37013 |
| Prevalence | Saudi Arabia | Male | 0.23537 | 1.60666 | -0.4042 |
| Prevalence | Saudi Arabia | Female | 0.550916 | 2.539992 | -0.32468 |
| Prevalence | Saudi Arabia | Both | 0.338926 | 1.805636 | -0.36027 |
| Prevalence | Libya | Male | 0.141511 | 1.216858 | -0.43055 |
| Prevalence | Libya | Female | 0.154437 | 1.651634 | -0.47005 |
| Prevalence | Libya | Both | 0.142571 | 1.093631 | -0.41755 |
| Prevalence | Syrian Arab Republic | Male | 0.236131 | 1.342093 | -0.36115 |
| Prevalence | Syrian Arab Republic | Female | 0.25654 | 1.613347 | -0.45618 |
| Prevalence | Syrian Arab Republic | Both | 0.24878 | 1.236311 | -0.32391 |
| Prevalence | Morocco | Male | 0.011576 | 0.76616 | -0.45293 |
| Prevalence | Morocco | Female | 0.18213 | 1.681139 | -0.43458 |
| Prevalence | Morocco | Both | 0.091476 | 0.897499 | -0.42429 |
| Prevalence | Bahrain | Male | 0.117012 | 1.058592 | -0.40835 |
| Prevalence | Bahrain | Female | 0.153244 | 1.627405 | -0.50175 |
| Prevalence | Bahrain | Both | 0.128704 | 1.0147 | -0.43078 |
| Prevalence | Sudan | Male | 0.335452 | 1.365039 | -0.2327 |
| Prevalence | Sudan | Female | 0.513707 | 2.012703 | -0.21972 |
| Prevalence | Sudan | Both | 0.424405 | 1.438892 | -0.19906 |
| Prevalence | Tunisia | Male | 0.028142 | 0.94293 | -0.48251 |
| Prevalence | Tunisia | Female | 0.113864 | 1.546207 | -0.51335 |
| Prevalence | Tunisia | Both | 0.070273 | 1.036461 | -0.45942 |
| Prevalence | Egypt | Male | 0.766391 | 2.340547 | -0.0777 |
| Prevalence | Egypt | Female | 1.042343 | 3.760179 | -0.16566 |
| Prevalence | Egypt | Both | 0.89434 | 2.776707 | -0.06918 |
| Prevalence | Palestine | Male | 0.226049 | 1.172294 | -0.34686 |
| Prevalence | Palestine | Female | 0.435563 | 2.262915 | -0.37674 |
| Prevalence | Palestine | Both | 0.325301 | 1.491257 | -0.31899 |
| Prevalence | TÃ¼rkiye | Male | 0.619815 | 1.917614 | -0.03709 |
| Prevalence | TÃ¼rkiye | Female | 0.636909 | 2.348947 | -0.24457 |
| Prevalence | TÃ¼rkiye | Both | 0.624876 | 1.923693 | -0.1057 |
| Incidence | Oman | Male | 0.032804 | 0.734593 | -0.37401 |
| Incidence | Oman | Female | 0.029223 | 0.699864 | -0.4044 |
| Incidence | Oman | Both | 0.020584 | 0.444011 | -0.3152 |
| Incidence | Iraq | Male | 0.340086 | 1.323963 | -0.12686 |
| Incidence | Iraq | Female | 0.466004 | 1.585242 | -0.11026 |
| Incidence | Iraq | Both | 0.397273 | 1.009607 | 0.002461 |
| Incidence | Yemen | Male | 0.046773 | 0.646938 | -0.37199 |
| Incidence | Yemen | Female | 0.183852 | 0.908183 | -0.27934 |
| Incidence | Yemen | Both | 0.102911 | 0.519947 | -0.20829 |
| Incidence | Qatar | Male | -0.36123 | 0.053813 | -0.62051 |
| Incidence | Qatar | Female | -0.33004 | 0.074826 | -0.62066 |
| Incidence | Qatar | Both | -0.34169 | -0.00481 | -0.5711 |
| Incidence | Jordan | Male | -0.20723 | 0.265743 | -0.51118 |
| Incidence | Jordan | Female | -0.28911 | 0.206407 | -0.58533 |
| Incidence | Jordan | Both | -0.24468 | 0.133473 | -0.49695 |
| Incidence | Saudi Arabia | Male | 0.006066 | 0.664273 | -0.37076 |
| Incidence | Saudi Arabia | Female | 0.309701 | 1.026964 | -0.19373 |
| Incidence | Saudi Arabia | Both | 0.104959 | 0.694851 | -0.31715 |
| Incidence | Egypt | Male | 0.612481 | 1.795974 | -0.13563 |
| Incidence | Egypt | Female | 0.836443 | 3.127543 | -0.18154 |
| Incidence | Egypt | Both | 0.715797 | 1.998641 | -0.10719 |
| Incidence | Iran (Islamic Republic of) | Male | 0.099163 | 0.773674 | -0.29587 |
| Incidence | Iran (Islamic Republic of) | Female | 0.026423 | 0.68966 | -0.42955 |
| Incidence | Iran (Islamic Republic of) | Both | 0.061842 | 0.609636 | -0.28101 |
| Incidence | Morocco | Male | -0.01099 | 0.512478 | -0.40201 |
| Incidence | Morocco | Female | -0.02742 | 0.591848 | -0.45082 |
| Incidence | Morocco | Both | -0.02057 | 0.317739 | -0.32084 |
| Incidence | TÃ¼rkiye | Male | 0.187731 | 0.894562 | -0.18814 |
| Incidence | TÃ¼rkiye | Female | 0.110828 | 0.727134 | -0.31719 |
| Incidence | TÃ¼rkiye | Both | 0.14741 | 0.560369 | -0.20518 |
| Incidence | Palestine | Male | -0.03282 | 0.473603 | -0.38354 |
| Incidence | Palestine | Female | 0.085309 | 0.835043 | -0.36234 |
| Incidence | Palestine | Both | 0.021641 | 0.480456 | -0.33102 |
| Incidence | United Arab Emirates | Male | -0.56242 | -0.23467 | -0.73497 |
| Incidence | United Arab Emirates | Female | -0.3694 | 0.040826 | -0.64477 |
| Incidence | United Arab Emirates | Both | -0.50295 | -0.30183 | -0.67502 |
| Incidence | Algeria | Male | 0.084846 | 0.670574 | -0.35245 |
| Incidence | Algeria | Female | 0.123342 | 0.813432 | -0.39589 |
| Incidence | Algeria | Both | 0.102244 | 0.525869 | -0.27896 |
| Incidence | Kuwait | Male | -0.34453 | -0.0876 | -0.52077 |
| Incidence | Kuwait | Female | -0.3617 | -0.04335 | -0.6025 |
| Incidence | Kuwait | Both | -0.3549 | -0.12724 | -0.55415 |
| Incidence | Syrian Arab Republic | Male | -0.04218 | 0.59821 | -0.42386 |
| Incidence | Syrian Arab Republic | Female | 0.005333 | 0.581044 | -0.40341 |
| Incidence | Syrian Arab Republic | Both | -0.02056 | 0.486651 | -0.3293 |
| Incidence | Lebanon | Male | 0.419676 | 1.529071 | -0.10349 |
| Incidence | Lebanon | Female | 0.347751 | 1.25268 | -0.20466 |
| Incidence | Lebanon | Both | 0.37316 | 1.122454 | -0.06434 |
| Incidence | Sudan | Male | 0.183732 | 0.959618 | -0.26136 |
| Incidence | Sudan | Female | 0.258015 | 1.101256 | -0.24228 |
| Incidence | Sudan | Both | 0.217294 | 0.710316 | -0.13641 |
| Incidence | Afghanistan | Male | 0.067632 | 0.70817 | -0.32537 |
| Incidence | Afghanistan | Female | 0.164793 | 0.886822 | -0.29678 |
| Incidence | Afghanistan | Both | 0.113708 | 0.554483 | -0.19452 |
| Incidence | Bahrain | Male | -0.13851 | 0.283597 | -0.45211 |
| Incidence | Bahrain | Female | -0.16938 | 0.288385 | -0.50079 |
| Incidence | Bahrain | Both | -0.15299 | 0.178793 | -0.41886 |
| Incidence | Tunisia | Male | -0.25096 | 0.195997 | -0.49598 |
| Incidence | Tunisia | Female | -0.25092 | 0.182207 | -0.53241 |
| Incidence | Tunisia | Both | -0.25412 | 0.061839 | -0.47413 |
| Incidence | Libya | Male | -0.09442 | 0.482524 | -0.43695 |
| Incidence | Libya | Female | -0.06363 | 0.624189 | -0.48168 |
| Incidence | Libya | Both | -0.08863 | 0.274173 | -0.39265 |
